# Evolution in French University Students' Mental Health One Month After the First COVID-19 Related Quarantine: Results From the COSAMe Survey

**DOI:** 10.3389/fpsyt.2022.868369

**Published:** 2022-05-03

**Authors:** Marielle Wathelet, Camille Vincent, Thomas Fovet, Charles-Edouard Notredame, Enguerrand Habran, Niels Martignène, Thierry Baubet, Guillaume Vaiva, Fabien D'Hondt

**Affiliations:** ^1^Univ. Lille, INSERM, Lille University Hospital (CHU de Lille), U1172—LilNCog - Lille Neuroscience & Cognition, Lille, France; ^2^Fédération de Recherche en Psychiatrie et Santé Mentale des Hauts-de-France (F2RSM), Lille, France; ^3^Centre National de Ressources et de Résilience Lille-Paris (CN2R), Lille, France; ^4^Department of Psychiatry, Lille University Hospital (CHU de Lille), Lille, France; ^5^Department of Public Health, Lille University Hospital (CHU de Lille), Lille, France; ^6^Fonds FHF Recherche et Innovation, Paris, France; ^7^Department of Infant, AP-HP, Avicenne Hospital, Child and Adolescent Psychiatry, Sorbonne Paris Nord, Paris, France

**Keywords:** COVID-19, pandemic (COVID19), quarantine, students, mental health

## Abstract

**Introduction:**

The COVID-19 related quarantine had negative psychological effects among University students. Evidence from previous epidemics suggests that negative psychological effects of quarantine measures can last or even worsen after the quarantine lift. The objective of this study was to assess the evolution of students' mental health and to identify factors associated with mental health outcomes 1 month after the lift of the lockdown.

**Materials and Methods:**

This repeated cross-sectional study collected data during the first quarantine in France (T1, *N* = 68,891) and 1 month after its lift (T2, *N* = 22,540), through an online questionnaire sent to all French University students. Using cross-sectional data, we estimated prevalence rates of suicidal thoughts, severe anxiety (State-Trait Anxiety Inventory, State subscale), depression (Beck Depression Inventory), and stress (Perceived Stress Scale) at T1 and T2. Using longitudinal data (*N* = 6,346), we identified risk factors of poor mental health outcomes among sociodemographic characteristics, precariousness indicators, health-related data, information on the social environment, and media consumption, adjusting for baseline mental health status.

**Results:**

We found lower prevalence rates of severe stress (21.7%), anxiety (22.1%), and depression (13·9%) one month after the quarantine compared to the quarantine period (24.8%, 27.5%, and 16.1%, respectively). The prevalence rate of suicidal thoughts increased from 11.4 to 13.2%. Regardless of the existence of symptoms during quarantine, four factors were systematically associated with poor mental health outcomes 1 month after the quarantine was lifted: female gender, a low feeling of integration before the quarantine period, a low quality of social ties during the quarantine, and a history of psychiatric follow-up.

**Conclusions:**

The prevalence rates of severe stress, anxiety, and depression, although being lower than during the first lockdown, remained high after its lift. The prevalence rate of suicidal ideation increased. This stresses the need to consider the enduring psychological impact of the pandemic on students as a critical public health issue.

## Introduction

On March 17, 2020, the French government mandated a quarantine on its territory, as many other countries did. This lockdown forbade all non-essential movements to limit the spread of the COVID-19 pandemic and lasted 8 weeks until May 11, 2020. If quarantine is one of the oldest tools to control contagious diseases, evidence from previous epidemics suggests that it also has a negative impact on the mental health of the population (1).

The negative psychological effects of the COVID-19 pandemic and related quarantine were rapidly confirmed (2), notably in University students, whose vulnerability to mental health problems is well-known (3). In France, the first measurement time (T1) of the repeated cross-sectional COSAMe study, conducted during the first lockdown period (from April 17 to May 4, 2020), revealed high prevalence rates of severe self-reported stress (24.7%), anxiety (27.5%), depression (16.1%), and suicidal thoughts (11.4%) among the 69,054 French University students who responded to the survey (4). Recently, a French study found that students reported more frequently perceived stress (33.1% vs 22.1%), anxiety (24.0% vs 14.7%), and depressive symptoms (32.5% vs 16.2%), as well as suicidal thoughts (11.7% vs 7.6%) than non-students during this period (5).

Evidence from previous epidemics suggests that the negative psychological effects of quarantine measures can last or even worsen after the quarantine lift (1). A recent review by Aknin et al. reports that, after an early peak, the psychological distress may have declined (6). While some studies showed a return to pre-pandemic levels after the first lockdown, other studies reported that the prevalence rates of mental health disorders, while being lower than those measured during the lockdown period, were still higher than estimates obtained outside any pandemic context (7, 8). The Lancet's COVID-19 Commission Mental Health Task Force recommends monitoring the mental health of populations over the next few years given the many warning signs that persist, beyond the first times of the pandemic (6). There are notably growing concerns about suicidal behavior (9, 10). If several sources demonstrate little change in suicide during the early months of the pandemic (6), the negative consequences of the pandemic on suicidal behavior could be delayed due to barriers to care access during the early stages of the pandemic (11), or due to the delayed economic consequences of the crisis, which are usually associated with an increase in suicidal behavior (12). France is particularly affected by suicide, with nearly 9,000 suicides per year. Suicide is also the second leading cause of death among young adults (15–24 years old), and the first warning signs have already been reported among this population: the consumption of anxiolytics, antidepressants, and hypnotics has increased during the first year of the COVID-19 pandemic, compared to the five previous years (13).

The present study used data from the COSAMe survey, including those collected during the second measurement interval (T2) 1 month after the first quarantine was lifted (from June 15 to July 15, 2020). At this time, the total number of confirmed cases of COVID-19 in France was nearly 173,000, and nearly 30,000 deaths were attributed to COVID-19. The aims of the study were: (i) measuring changes in prevalence rates of self-reported mental health symptoms (stress, anxiety, depression, and suicidal thoughts) using repeated cross-sectional data, and (ii) identifying factors associated with mental health outcomes 1 month after the lift of the lockdown, adjusting for baseline mental health status, using longitudinal data.

## Materials and Methods

### Study Design and Study Population

To promote student participation at each measurement interval of the COSAMe survey, the French Ministry of Higher Education, Research, and Innovation asked all 82 French universities to offer their students the opportunity to complete an online questionnaire sent by email (target population: approximately 1,600,000 students). The first measurement time (T1) took place during the COVID-19 lockdown, between April 17 and May 4, 2020. The second measurement time (T2) occurred 1 month after the quarantine was lifted between June 15 and July 15, 2020. The eligibility criteria were being a University student and having resided in France during the first lockdown. Students who answered both T1 and T2 were linked using a pseudonymization method.

The CHERRIES checklist, recommended for reporting the results of Internet e-surveys, is available in Supplementary Table 1 (14).

This study was approved by a French research ethics committee, the *Comité de Protection des Personnes Ile de France VIII*, before its initiation. The protocol of COSAMe and detailed results of T1 have been published elsewhere (4).

### Data Collected

#### Outcomes

We focused on the following 4 outcomes, collected at T1 and T2:

i suicidal thoughts, by asking participants whether they had experienced suicidal thoughts during the preceding month,
ii depression, using the validated French version of the 13-item Beck Depression Inventory (BDI-13) (15),
iii anxiety, through the validated French version of the 20-item State-Trait Anxiety Inventory, State subscale (STAI Y-2) (16),
iv stress, using the validated French version of the Perceived Stress Scale (PSS-10) (17).

Outcomes were the presence of severe self-reported symptoms, i.e., the presence of suicidal thoughts or a high score on at least 1 scale, as defined in the literature (i.e., PSS-10 >26; BDI-13 >15; or STAI-Y2 >55).

#### Covariates

Regarding covariates, we considered (i) sociodemographic characteristics (gender, year of study, being a foreign student, living area, living in a worst-hit department), (ii) economic indicators (housing quality, loss of income due to quarantine), (iii) health-related information (history of psychiatric follow-up, symptoms consistent with COVID-19 since the beginning of the pandemic, and physical activity during the quarantine), (iv) media or information data (consumption of media information related to the pandemic in minutes per day and perceived quality of information received), and (v) social support indicators (feeling socially integrated before the quarantine, having children, housing composition during the quarantine, concern for relatives' health, quality of perceived social relationships during the quarantine).

### Statistical Analysis

Only students for whom full data was available were analyzed. The statistical analyses were conducted in three stages.

The first analysis described the crude prevalence rates of mental health outcomes at each measurement time as the number of prevalent cases divided by the total number of respondents. Gender- and degree-standardized prevalence rates were calculated using the University Students population 2019–2020 published by the French Ministry of National Education (18). These standardized rates were calculated excluding non-binary students since there is no available information regarding their proportion among students.

The second analysis assessed the association between quarantine and mental health outcomes. Bivariate analyses using Chi-2 tests compared proportions of mental health disorders at T2 (after quarantine) and T1 (during quarantine). Then, we carried out logistic regression models to assess the impact of no longer being quarantined (T2 vs. T1) on mental health, after adjustment for all covariates described in the previous section.

In the third analysis, only students who answered both T1 and T2 questionnaires were considered. To identify factors associated with mental health outcomes at T2, we performed multivariate logistic regression models for each outcome. Models were adjusted for all covariates, including mental health status at baseline (score above the threshold at T1 for stress, anxiety, and depression, or presence of suicidal thoughts at T1 for suicidal thoughts). Moreover, associations between suicidal ideation and other mental health disorders at T2 have also been studied using Chi-square tests.

Data analysis was performed using R version 3.4.2. The significance level was set at α = 0.05 and all tests were 2-tailed. Results of regression models are presented as adjusted prevalence odds ratios and 95% confidence intervals (aOR [95%CI]).

## Results

### Repeated Cross-Sectional Data

In total, 96,861 students opened the questionnaire at T1, and 28,120 at T2. A total of 68,891 students fully completed the questionnaire at T1 and 22,540 at T2 (response rate: 4.2% of French University students at T1 and 1.3% at T2). The vast majority of the sample was made up of bachelor students at both T1 and T2 (80.4% and 77.4%, respectively). Women were over-represented whatever the degree (72.7% at T1, 72.5% at T2), and more than half of the sample were female bachelor students (58.8% at T1, and 56.4% at T2). Non-binary students represented 1.1% of the sample at T1 (*N* = 785), and 1.5% at T2 (*N* = 331).

Crude and standardized prevalence rates are described in Table 1. A significant (*p* < 0.001) lower proportion of severe self-reported stress, anxiety and depression was measured at T2 [standardized prevalence rates (95%CI) at 20.1% vs. 22.4%, 20.8% vs. 25.5%, and 12.6% vs. 14.3%, respectively]. However, the proportion of suicidal thoughts increased (12.3% vs. 10.6%, *p* < 0.001).

**Table 1:** Crude and adjusted prevalence rates of mental health outcomes at T1 and T2 in the whole sample (*n* = 68,891 at T1; *n* = 22,540 at T2) and detailed prevalence rates according to gender and degree.

	**Characteristics**			**Mental health outcomes**							
	**Target population**	**Study sample**		**Stress (PSS >26)**		**Anxiety (STAI-Y2 >55)**		**Depression (BDI >15)**		**Suicidal thoughts**	
		**T1**	**T2**	**T1**	**T2**	**T1**	**T2**	**T1**	**T2**	**T1**	**T2**
**Crude prevalence rate** [95%CI] *including non-binary students*		*N* = 68,891	*N* = 22,540	24.8 [24.4–25.1]	21.7 [21.2–22.3]	27.5 [27.1–27.8]	22.1 [21.6–22.7]	16.1 [15.8–16.4]	13.9 [13.5–14.4]	11.4 [11.2–11.7]	13.2 [12.7–13.6]
**Crude prevalence rate** [95%CI] *excluding non-binary students*		*N* = 68,106	*N* = 22,205	24.5 [24.2–24.8]	21.4 [20.9–22.0]	27.2 [26.9–27.5]	21.8 [21.2–22.3]	15.8 [15.5–16.1]	13.7 [13.2–14.1]	11.0 [10.8–11.3]	12.7 [12.3–13.2]
**Standardized prevalence rate** [95%CI]	*N* = 1,635,350	*N* = 68,106	*N* = 22,205	22.4 [22.1–22.7]	20.1 [19.6–20.6]	25.5 [25.2–25.8]	20.8 [20.3–21.4]	14.3 [14.1–14.6]	12.6 [12.2–13.1]	10.6 [10.3–10.8]	12.3 [11.8–12.7]
**Bachelor**, n (%)											
Men	423,923 (25.9)	14,250 (20.9)	4,462 (20.1)	2,071 (14.5)	633 (14.2)	2,349 (16.5)	643 (14.4)	1,702 (11.9)	520 (11.6)	1,425 (10.0)	521 (11.7)
Women	573,542 (35.1)	40,521 (59.5)	12,717 (57.3)	11,430 (28.2)	3,014 (23.7)	12,370 (30.5)	3,001 (23.6)	7,347 (18.1)	1,971 (15.5)	4,744 (11.7)	1,713 (13.5)
**Master**, n (%)											
Men	234,829 (14.3)	3,266 (4.8)	1,207 (5.4)	515 (15.8)	176 (14.6)	652 (20.0)	215 (17.8)	339 (10.4)	120 (9.9)	298 (9.1)	134 (11.1)
Women	347,872 (21.3)	8,658 (12.7)	3,217 (14.5)	2,419 (27.9)	819 (25.5)	2,811 (32.5)	845 (26.3)	1,265 (14.6)	371 (11.5)	897 (10.4)	394 (12.2)
**Doctorate**, n (%)											
Men	28,365 (1.7)	463 (0.7)	187 (0.8)	59 (12.7)	24 (12.8)	80 (17.3)	32 (17.1)	35 (7.6)	14 (6.9)	55 (11.9)	15 (8.0)
Women	26,819 (1.6)	948 (1.4)	415 (1.9)	195 (20.6)	97 (23.4)	270 (28.5)	96 (23.1)	82 (8.6)	41 (9.9)	93 (9.8)	46 (11.1)

After adjustment, identical patterns were identified (Table 2): no longer being quarantined (T2 vs. T1) was significantly associated with a lower risk of severe self-reported stress [OR (95%CI) = 0.81 (0.77–0.86)], anxiety [0.74 (0.69–0.78)] and depression [0.81 (0.76–0.87)]. However, it was associated with a significantly higher risk of suicidal thoughts [1.13 (1.05–1.21)].

**Table 2:** Association between no longer being quarantined (T2 vs. T1) and mental health outcomes, in the global and the paired samples: results of bivariate and multivariate analyzes.

	**Stress (PSS >26)**	**Anxiety (STAI-Y2 >55)**	**Depression (BDI >15)**	**Suicidal thoughts**
**Global sample**				
Crude OR [95%CI]	0.84 [0.81–0.87]	0.75 [0.72–0.78]	0.84 [0.81–0.88]	1.17 [1.12–1.23]
Adjusted OR [95%CI]	0.81 [0.77–0.86]	0.74 [0.69–0.78]	0.81 [0.76–0.87]	1.13 [1.05–1.21]
**Paired sample**				
OR [95%CI]	0.67 [0.59–0.76]	0.70 [0.62–0.79]	0.89 [0.76–1.05]	1.34 [1.12–1.61]

### Longitudinal Data

#### Mental Health Outcomes at T1 and T2

Characteristics of the paired sample (*N* = 6,346) are reported in Table 3. When considering only the students who answered both T1 and T2, we found similar prevalence rates of mental health outcomes to those observed in the full sample. At T1, among the 6,346 students who both answered T1 and T2, 795 (12.5%) reported suicidal thoughts, 1,653 (26.0%) severe stress, 1,658 (26.1%) severe anxiety, and 933 (14.7%) severe depression. At T2, they were 869 (13.7%), 1,432 (22.6%), 1,465 (23.1%), 900 (14.2%), respectively. For the association between the lift of quarantine and mental health outcomes, patterns were similar to those found in the overall sample, although not significant for depression (Table 2).

**Table 3:** Factors associated with severe symptoms at T2 (*n* = 6,346): results of the multivariate logistic regression models.

	**Characteristics**	**PSS-10 > 26 at T2**		**STAI-Y2 > 55 at T2**		**BDI-13 > 15 at T2**		**Suicidal thoughts at T2**	
	**Respondents at T1 and T2**	**aOR [95%CI]^**‡**^**	**p^**§**^**	**aOR [95%CI]^**‡**^**	**p^**§**^**	**aOR [95%CI]^**‡**^**	**p^**§**^**	**aOR [95%CI]^**‡**^**	**p^**§**^**
Baseline mental health status^¶^		9.7 [8.4–11.3]	**<0.001**	10.5 [9.0–12.2]	**<0.001**	21.2 [17.4–25.9]	**<0.001**	33.8 [27.5–41.8]	**<0.001**
**Demographic characteristics**									
**Gender**, n (%)			**0.001**		**0.003**		0.104		**0.046**
Male	1,442 (22.7)	ref		ref		ref		ref	
Female	4,795 (75.6)	1.38 [1.14–1.68]	**0.001**	1.37 [1.13–1.66]	**0.001**	1.30 [1.02–1.68]	**0.037**	1.30 [1.01–1.67]	**0.042**
Non-binary	109 (1.7)	1.85 [1.12–3.03]	**0.015**	1.68 [1.02–2.77]	**0.039**	1.12 [0.60–2.05]	0.713	1.82 [0.99–3.30]	0.051
**Year of study**, n (%)			0.539		**0.040**		0.378		0.223
Bachelor	4,852 (76.5)	ref		ref		ref		ref	
Master	1,296 (20.4)	1.10 [0.92–1.32]	0.291	1.24 [1.03–1.48]	**0.021**	0.93 [0.72–1.19]	0.569	0.90 [0.70–1.16]	0.429
Doctorate	198 (3.1)	1.12 [0.72–1.69]	0.615	0.85 [0.54–1.30]	0.464	0.65 [0.33–1.21]	0.197	0.61 [0.32–1.10]	0.113
**Foreign student** (Yes vs No), n (%)	218 (3.4)	0.87 [0.58–1.27]	0.476	1.31 [0.89–1.91]	0.161	1.50 [0.92–2.40]	0.103	0.44 [0.23–0.81]	**0.007**
**Department of residence affected** (Yes vs No), n (%)	1,850 (29.1)	0.96 [0.81–1.12]	0.588	1.03 [0.88–1.21]	0.675	1.00 [0.81–1.24]	0.966	1.02 [0.82–1.26]	0.869
**Area**, n (%)			0.074		0.420		0.290		0.519
Urban	2,920 (46.0)	ref		ref		ref		ref	
Semiurban	1,658 (26.1)	1.15 [0.96–1.38]	0.586	1.03 [0.86–1.24]	0.739	1.19 [0.94–1.51]	0.141	1.09 [0.85–1.40]	0.483
Rural	1,768 (27.9)	0.92 [0.76–1.11]	0.374	0.91 [0.75–1.09]	0.309	1.15 [0.90–1.48]	0.258	0.93 [0.72–1.21]	0.600
**Precariousness indicators**									
**Loss of income** (Yes vs No), n (%)	1,071 (16.9)	1.13 [0.94–1.36]	0.195	1.03 [0.85–1.24]	0.755	1.15 [0.91–1.46]	0.246	1.31 [1.02–1.67]	**0.035**
**Housing quality** (rated out of 10), n (%)			0.167		**0.036**		0.662		0.830
High (7-10)	5,504 (86.7)	ref		ref		ref		ref	
Medium (4-6)	705 (11.1)	1.23 [0.99–1.53]	0.058	1.32 [1.07–1.64]	**0.010**	1.13 [0.86–1.48]	0.370	0.92 [0.68–1.23]	0.587
Low (0-3)	137 (2.2)	1.07 [0.68–1.66]	0.765	0.98 [0.63–1.53]	0.931	0.99 [0.58–1.68]	0.979	0.90 [0.49–1.62]	0.724
**Social data**									
**Having children** (Yes vs No), n (%)	69 (1.1)	0.65 [0.29–1.35]	0.260	0.77 [0.36–1.57]	0.488	0.71 [0.23–1.83]	0.495	0.72 [0.21–2.01]	0.561
**Housing arrangement**, n (%)			**0.023**		0.152		0.448		0.489
Living with family	5,262 (82.9)	ref		ref		ref		ref	
Living alone	773 (12.2)	1.24 [0.98–1.56]	0.071	1.23 [0.98–1.55]	0.077	1.20 [0.89–1.61]	0.221	1.10 [0.80–1.49]	0.550
Living with roommates	224 (3.5)	1.07 [1.22–1.59]	0.734	1.22 [0.82–1.79]	0.313	0.81 [0.45–1.41]	0.478	1.34 [0.79–2.21]	0.260.
Other	87 (1.4)	2.16 [0.71–3.72]	**0.006**	1.55 [0.85–2.75]	0.145	0.82 [0.38–1.69]	0.594	1.52 [0.70–3.12]	0.273
**Feeling of integration**, n (%)			**<0.001**		**<0.001**		**<0.001**		**<0.001**
High (7–10)	4,037 (63.6)	ref		ref		ref		ref	
Medium (4–6)	1,872 (29.5)	1.34 [1.14–1.57]	**<0.001**	1.78 [1.52–2.08]	**<0.001**	2.42 [1.97–2.98]	**<0.001**	1.86 [1.50–2.29]	**<0.001**
Low (0–3)	437 (6.9)	2.43 [1.86–3.17]	**<0.001**	2.54 [1.94–3.32]	**<0.001**	4.40 [3.22–6.02]	**<0.001**	2.76 [1.98–3.85]	**<0.001**
**Concern for relatives' health** (rated out of 10), n (%)			**<0.001**		**<0.001**		**0.001**		0.700
Low (0–3)	1,040 (16.4)	ref		ref		ref		ref	
Medium (4–6)	2,050 (32.3)	1.07 [0.84–1.37]	0.562	0.93 [0.73–1.19]	0.590	0.91 [0.37–1.26]	0.594	1.11 [0.82–1.50]	0.496
High (7–10)	3,256 (51.3)	1.63 [1.30–2.05]	**<0.001**	1.30 [1.04–1.63]	**0.022**	1.36 [1.02–1.82]	**0.034**	1.13 [0.85–1.51]	0.406
**Quality of social ties** (rated out of 10), n (%)			**0.005**		**<0.001**		**0.002**		**0.005**
High (7–10)	2,847 (44.9)	ref		ref		ref		ref	
Medium (4–6)	2,475 (39.0)	1.15 [0.98–1.36]	0.079	1.35 [1.15–1.59]	**<0.001**	1.25 [1.00–1.56]	**0.047**	1.44 [1.16–1.80]	**<0.001**
Low (0–3)	1,024 (16.1)	1.40 [1.14–1.72]	**0.001**	1.39 [1.12–1.71]	**0.002**	1.59 [1.23–2.06]	**<0.001**	1.21 [0.91–1.59]	0.183
**Media and information**									
**Time spent consulting information** (in min/d), n (%)			0.273		0.215		**0.022**		**0.011**
<15	2,355 (37.1)	ref		ref		ref		ref	
15–29	1,110 (17.5)	0.90 [0.73–1.12]	0.370	0.88 [0.71–1.09]	0.260	0.97 [0.73–1.29]	0.863	0.70 [0.52–0.94]	**0.020**
30–59	1,339 (21.1)	1.02 [0.83-1.24]	0.854	0.97 [0.79-1.18]	0.748	0.78 [0.60-1.02]	0.071	0.72 [0.54-0.95]	**0.020**
60–119	1,066 (16.8)	1.06 [0.86–1.31]	0.595	1.05 [0.84–1.29]	0.671	1.11 [0.84–1.45]	0.469	1.01 [0.76–1.35]	0.917
≥120	476 (7.5)	1.27 [0.97-1.66]	0.081	1.26 [0.96–1.65]	0.096	1.44 [1.02–2.02]	**0.037**	1.18 [0.82–1.69]	0.359
**Quality of information received** (rated out of 10), n (%)			**<0.001**		**0.002**		0.313		0.879
High (7–10)	2,461 (38.8)	ref		ref		ref		ref	
Medium (4–6)	2,889 (45.5)	1.26 [1.07–1.48]	**0.005**	1.14 [0.97–1.34]	0.119	1.08 [0.87–1.33]	0.489	1.04 [0.84–1.29]	0.727
Low (0–3)	996 (15.7)	1.47 [1.19–1.82]	**<0.001**	1.47 [1.19–1.81]	**<0.001**	1.24 [0.94–1.62]	0.126	1.07 [0.81–1.42]	0.627
**Health-related data**									
**History of psychiatric follow-up** (Yes vs. No), n (%)	778 (12.2)	1.75 [1.44–2.12]	**<0.001**	1.77 [1.46–2.16]	**<0.001**	1.92 [1.51–2.42]	**<0.001**	2.14 [1.68–2.72]	**<0.001**
**Symptoms consistent with COVID-19** (Yes vs. No), n (%)	2023 (31.9)	1.61 [1.34–1.94]	**<0.001**	1.62 [1.34–1.95]	**<0.001**	1.23 [0.96–1.56]	0.095	1.35 [1.05–1.73]	**0.019**
**Duration of physical activity** (in min/d), n (%)			**0.017**		0.939		0.829		0.285
≥60	1,887 (29.7)	ref		ref		ref		ref	
30–59	1,824 (28.7)	1.12 [0.93–1.36]	0.229	1.01 [0.83–1.19]	0.922	0.95 [0.74–1.23]	0.702	0.96 [0.73–1.24]	0.580
15–29	1,205 (19.0)	1.00 [0.81–1.25]	0.963	1.05 [0.84–1.30]	0.669	1.05 [0.80–1.40]	0.706	1.25 [0.93–1.66]	0.207
<15	1,430 (22.5)	1.34 [1.10–1.64]	**0.004**	0.98 [0.80–1.22]	0.816	0.93 [0.72–1.21]	0.599	1.10 [0.84–1.45]	0.594

‡ *aOR [95%CI] = adjusted odd ratio [95% confidence interval]*.

§ *For each variable, the p-value opposite the name of the variable refers to its global effect, and when applicable, p-value referring to each category vs reference are also presented*.

¶ *Severe symptoms already reported at baseline (i.e., PSS-10 > 26 at T1 for stress, STAI-Y2 > 55 at T1 for anxiety, BDI-13 > 15 at T1 for depression, and suicidal thoughts at T1 for suicidal thoughts)*.

*Bold values correspond to significant associations (p-values < 0.05)*.

#### Factors Associated With Mental Health Symptoms at T2

Factors associated with mental health outcomes at T2 are presented in Table 3. Excepted mental health status at baseline, four factors were significantly associated with all poor mental health outcomes at T2: female gender, low feeling of integration, low quality of social ties, and history of psychiatric follow-up. Concerning gender, aOR [95%CI] for female gender (vs male gender) was between 1.30 [1.01–1.67], *p* = 0.042 (for suicidal thoughts) and 1.38 [1.14–1.68], *p* < 0.001 (for stress). Non-binary gender was significantly at risk for stress and anxiety. The lower the feeling of integration, the more students were at risk (aOR [95%CI] from 2.43 [1.86–3.17], *p* < 0.001 for stress, to 4.40 [3.22–6.02], *p* < 0.001 for depression). Concerning the quality of social ties during quarantine, students were more at risk when they reported a lower quality (aOR [95%CI] from 1.39 [1.12–1.71], *p* = 0.002 for anxiety, to 1.59 [1.23–2.06], *p* < 0.001 for depression). This pattern was not as marked for suicidal ideation. Finally, compared to students without psychiatric history, those who reported a history of psychiatric follow-up were at higher risk of mental health outcomes [from 1.75 (1.44–2.12), p < 0.001 for stress, to 2.14 (1.68–2.72), *p* < 0.001 for suicidal thoughts].

Other factors were not associated with all mental health outcomes. Concerning demographic variables, students in master's degree were more at risk of anxiety than students in bachelor's degree [aOR (95%CI] = 1.24 [1.03–1.48), *p* = 0.021], and foreign students were less at risk of suicidal thoughts [0.44 (0.23–0.81), *p* = 0.007]. Regarding precarity, a loss of income due to the COVID-19 pandemic was at risk of suicidal thoughts at T2 [1.31 (1.02–1.67), *p* = 0.035]. An association was also found between anxiety and medium housing quality, compared to high quality [1.32 (1.07–1.64), *p* = 0.010]. But there was no association with low housing quality. Among social variables, a high level of concern for relatives' health was at risk of stress [1.63 (1.30–2.05), *p* < 0.001], anxiety [1.30 (1.04–163), *p* = 0.022], and depression [1.36 (1.02–1.82) at T2], but no association was found with suicidal thoughts. Concerning media and information, low quality of the information received was associated with a higher risk of stress [1.47 (1.19–1.82), *p* < 0.001], and anxiety [1.47 (1.19–1.81), *p* < 0.001]. Media consumption for more than 2 h per day, compared to <15 min, was associated with an increased risk of depression [1.44 (1.02–2.02), *p* = 0.037], while moderate consumption (between 15 min and an hour) was protective of suicidal ideation [0.70 (0.52–0.94), *p* = 0.020 for 15–29 min, and 0.72 (0.54–0.95), *p* = 0.020 for 30–59 min]. Finally, regarding health-related variables, having experienced symptoms consistent with COVID-19 was significantly associated with stress, anxiety and suicidal thoughts at T2 [1.61 (1.34–1.94], *p* < 0.001, 1.62 (1.34–1.95), *p* < 0.001 and 1.35 (1.05–1.73), *p* = 0.019, respectively]. The year of study was significantly associated with worsening anxiety and depression, but patterns were unclear. A low practice of physical exercise (<15 min per day vs. more than 60 min), was associated with an increased risk of stress [1.34 (1.10–1.64), *p* = 0.004].

#### Association Between Suicidal Ideation and Other Mental Health Symptoms at T2

Finally, the prevalence rates of suicidal thoughts at T2 were significantly higher in students whose symptoms were severe at T2 (Table 4). Among students who reported severe symptoms of stress or anxiety, more than a third also reported suicidal ideation (38.5% vs. 6,5%, *p* < 0.001 for stress, and 38.6% vs. 6.2%, *p* < 0.001 for anxiety). This proportion exceeded 50% for those reporting severe symptoms of depression (57.6% vs. 6.4%, *p* < 0.001).

**Table 4:** Association between suicidal thoughts and severe symptoms of stress, anxiety and depression at T2 (*n* = 6,346): results of the bivariate analyses.

	**PSS-10 > 26 at T2**		**STAI-Y2 > 55 at T2**		**BDI-13 > 15 at T2**	
	**Yes**	**No**	**Yes**	**No**	**Yes**	**No**
	***N* = 1,432**	***N* = 4,914**	***N* = 1,465**	***N* = 4,881**	***N* = 900**	***N* = 5,446**
**Suicidal thoughts at T2**						
Yes	551 (38.5)	318 (6.5)	565 (38.6)	304 (6.2)	518 (57.6)	351 (6.4)
No	881 (61.5)	4,596 (93.5)	900 (61.4)	4,577 (93.8)	382 (42.4)	5,095 (93.6)
p	**< 0.001**		**< 0.001**		**< 0.001**	

*Bold values correspond to significant associations (p-values < 0.001)*.

## Discussion

This study revealed lower prevalence rates of severe symptoms of stress (21.7%), anxiety (22.1%), and depression (13.9%) among University students 1 month after the COVID-19 related quarantine was lifted in France when compared to the quarantine period (24.8%, 27.5%, and 16.1% for severe self-reported stress, anxiety, and depression, respectively). Conversely, the prevalence rate of suicidal thoughts increased from 11.4% of the students during the quarantine, to 13.2% 1 month after the quarantine was ended. Overall, four factors, which had already been identified as risk factors for mental health disorders during the quarantine (4), were significantly associated with poor mental health outcomes (suicidal thoughts, severe symptoms of anxiety, depression, and stress) 1 month after the COVID-19 related quarantine was lifted: female gender, a low feeling of integration, low quality of social ties, and a history of psychiatric follow-up.

Regarding anxiety and depression, our results are consistent with the decrease in prevalence rates obtained by Lu et al. (19) in the only study that compared prevalence rates of mental health symptoms during and after a COVID-19 related lockdown period, although using different samples (19). In line with the particular vulnerability of the University student population to the psychological impact of the quarantine, we found higher prevalence rates of severe self-reported symptoms of anxiety and depression (22.1% and 13.9%, respectively) than Lu et al. (6.3 and 6.8%, respectively) who conducted their study in the Chinese general population. Importantly, although these prevalence rates were lower 1 month after than during the lockdown, they remained higher than before the COVID-19 pandemic. Indeed, a study involving 4,184 French undergraduate University students in 2017 reported prevalence rates of 12.6% and 7.6%, for depression and anxiety, respectively (20).

Regarding suicidal ideations, several studies found high rates of suicidal ideations during COVID-19 related quarantine (4, 21) but the present study is the first to assess and show increased suicidal ideation after a quarantine lift. This result is in line with observations made by the *French Institute of Public Opinion* (IFOP), which found that 17% of the participants with lifetime suicidal ideations reported having experienced them after the quarantine, compared to 11% during the quarantine (overall 20% of the sample reported lifetime suicidal ideation) in a survey of 2,000 participants, representative of the French population aged 18 and over, carried out in September 2021 (22). Importantly, we found more frequent suicidal ideations among students who reported other severe mental health symptoms after the lift of the quarantine, which is consistent with the high prevalence of suicidal ideations in people affected by psychiatric symptoms and disorders (23), including during pandemic crises (24). Loss of income, feeling of integration, and quality of social ties, which are well-documented predisposing factors for suicide (12, 23), were also significantly associated with an increased risk of reporting suicidal thoughts after quarantine. The female gender is also a well-known risk factor for depression, anxiety, stress, and suicidal ideation in the general population (25, 26). Among University students, while literature is inconclusive concerning depression, many studies have also shown that female students are vulnerable to these disorders (27). Beyond genetic and biological factors, the pandemic context may have reinforced inequalities, including those based on gender, as has been shown in previous epidemics (28). Furthermore, although the sample is smaller and subject to a lack of power, the proportions of mental health disorders among non-binary participants were particularly high and should be the subject of in-depth studies.

Considering that stressful life events, including natural disasters, precede many suicides and suicide attempts (23) and because suicidal ideation is an indicator of future suicide attempts, psychiatric disorders, and global impaired functioning (29–32), particular attention should be paid to the high prevalence rates of suicidal ideation in the student population during the COVID-19 pandemic. Moreover, recent estimations predict an increase in the number of suicides based on the expected number of job losses due to COVID-19 (12). Several recommendations have been formulated using examples from countries that have efficiently broken the link between unemployment and suicide rates and notably promote: (i) access to secondary prevention (treat disorders such as depression), (ii) active labor market programs, and (iii) gender equality in the workplace (33).

Some limitations should be considered in the interpretation of these results. First, although the number of respondents is large, it represents 4.2% of French University students at T1 and 1.3% at T2. Caution is therefore necessary before generalizing these results. Nevertheless, this problem is encountered in all large epidemiologic studies and does not systematically mean that a self-selection bias has altered the results (34, 35). Indeed, it has been shown that a low response rate in epidemiological surveys only marginally affects prevalence and association measures (34, 35). Besides, the prevalence rates were stratified by and adjusted for gender and degree, and multivariate analyses included gender and degree as a covariate. To control for potential confounding bias related to differences in sample characteristics from one measurement time to another, the multivariate models included all covariates, and a subgroup analysis on the longitudinal data was performed. Second, although validated, the questionnaires used for this study to identify mental health symptoms are screening but not diagnostic tools. However, a high score on these validated tests is highly correlated with the presence of a mental health disorder. Finally, the questionnaire did not include other risk factors that could be associated with suicidal ideation, such as substance use disorder or personal or family history of suicidal behavior (36).

As a whole, two main conclusions can be drawn from the present study. First, severe symptoms of stress, anxiety, and depression were less prevalent after than during the COVID-19 related quarantine among University students but remained more prevalent than before the pandemic. Second, suicidal ideations, which were already frequent during the quarantine, were even more prevalent after the lift of the quarantine. This stresses the need to consider the psychological impact of the pandemic on students as a critical public health issue, demanding an urgent and strong policy response. Future studies will necessarily have to assess the long-term consequences of this enduring crisis, with a special focus on suicidal behavior.

## Data Availability Statement

The raw data supporting the conclusions of this article will be made available by the authors, without undue reservation.

## Ethics Statement

The studies involving human participants were reviewed and approved by Comité de Protection des Personnes Ile de France VIII. Written informed consent for participation was not required for this study in accordance with the national legislation and the institutional requirements.

## Author Contributions

MW, GV, and FD'H contributed to the conception and design of the study. MW, CV, NM, and EH organized the database. CV and MW performed the statistical analysis. MW and FD'H wrote the first draft of the manuscript. All authors contributed to manuscript revision, read, and approved the submitted version.

## Conflict of Interest

The authors declare that the research was conducted in the absence of any commercial or financial relationships that could be construed as a potential conflict of interest.

## Publisher's Note

All claims expressed in this article are solely those of the authors and do not necessarily represent those of their affiliated organizations, or those of the publisher, the editors and the reviewers. Any product that may be evaluated in this article, or claim that may be made by its manufacturer, is not guaranteed or endorsed by the publisher.
