# Supplementary material for: Evolution in French University Students' Mental Health One Month After the First COVID-19 Related Quarantine: Results From the COSAMe Survey
Source: Front Psychiatry. 2022 May 3;13:868369. doi: 10.3389/fpsyt.2022.868369 (PMC9110762; doi:10.3389/fpsyt.2022.868369)
Supplement: Supplementary file 1 [file Table_1.DOCX]

**Supplementary table 1: The CHERRIES Checklist for the two first measurement times of the COSAMe survey**

| ***Item Category*** | ***Checklist Item*** | ***Explanation*** |
| --- | --- | --- |
| **Design** |  |  |
|  | Describe survey design | The target population was students enrolled at university in France during the 2019-2020 school year (N = approximately 1,600,000 students).  The first measurement time (T1) took place during the COVID-19 lockdown, between April 17 and May 4, 2020. The second measurement time (T2) occurred one month after the quarantine was lifted between June 15 and July 15, 2020.  To promote student participation in this "open" survey, the French Ministry of Higher Education, Research, and Innovation asked the 82 French universities to inform their students by email of the opportunity to fill in an online questionnaire, at each measurement time of the COSAMe survey. |
| **IRB (Institutional Review Board) approval and informed consent process** |  |  |
|  | IRB approval | This study was approved by a French research ethics committee (the *Comité de Protection des Personnes Ile de France VIII*). |
|  | Informed consent | Oral or written consent was not required for this study because responding to the survey was considered consent to participate.  In an information note on the introduction page of the survey, the students were informed about how long it would take to complete the questionnaire, what data would be stored, where and for how long. Finally, the purpose of the study and the name of the investigator were explicit. |
|  | Data protection | The survey was anonymous but the responses between T1 and T2 have been linked using a pseudonymization method. The data were securely stored with a secure site of the French Hospital Federation (FHF). |
| **Development and pre-testing** |  |  |
|  | Development and testing | The questionnaire was developed based on a review of the literature. A group of experts then selected the relevant variables and the appropriate and validated tools according to the judgment criteria chosen.  The questionnaire was published online by the Research & Innovation Fund of the French Hospital Federation and tested by the investigators and the students associated with the research team. |
| **Recruitment process and description of the sample having access to the questionnaire** |  |  |
|  | Open survey versus closed survey | This was an "open survey" but the URL link to access the questionnaire was sent by the universities only to students, to minimize the participation of non-students.  At the beginning of the questionnaire, it was also reminded that the questionnaire was for students only.  Only fully completed questionnaires were retained for analysis. |
|  | Contact mode | Initial contact with potential participants was made by email. The email contained the information note and the URL link to access the questionnaire. |
|  | Advertising the survey | The university communication services, and the French National Center for School and University Affairs (CNOUS) informed their university students by mail of the opportunity to participate in the survey. |
| **Survey administration** |  |  |
|  | Web/E-mail | An online questionnaire captured the responses. |
|  | Context | The site has been specially created for PTSD studies in the COVID-19 pandemic context by the Research & Innovation Fund of the Fédération Hospitalière de France (FHF). The URL of the site has been distributed by email to students only (with no pre-selection process). |
|  | Mandatory/voluntary | It was a voluntary survey. |
|  | Incentives | No compensation was offered. The results of the studies were published on the CN2R website. |
|  | Time/Date | The first measurement time (T1) took place during the first COVID-19 lockdown, between April 17 and May 4, 2020. The second measurement time (T2) occurred one month after the quarantine was lifted between June 15 and July 15, 2020. |
|  | Randomization of items or questionnaires | There was no randomization of items. |
|  | Adaptive questioning | Conditional items were conditionally displayed based on responses to other items, and appeared only for concerned participants. |
|  | Number of Items | The total number of items was 158.  The number of questionnaire items per page was approximately 20. |
|  | Number of screens (pages) | The questionnaire consisted of 8 pages. |
|  | Completeness check | Responses were proposed for each item. For items requiring a free field, the format was controlled (eg, numerical response for quantitative variables, such as age). To move to the next page, the student had to have completed all of the items. At each change of page, the data was saved. |
|  | Review step | Once the page change was made, it was not possible to go back. |
| **Response rates** |  |  |
|  | Unique site visitor | We did not identify a unique user on the site (only the number of visits was collected). An identifier was not assigned until the student agreed to participate in the survey.  A unique identifier was defined for each participant based on 2 personal but non-identifying questions (name of preferred teacher, and last 5 digits of the phone number). |
|  | View rate (Ratio of unique survey visitors/unique site visitors) | The website was directly dedicated to the study. Thus, all site visitors are survey visitors. |
|  | Participation rate (Ratio of unique visitors who agreed to participate/unique first survey page visitors) | T1  visitors who agreed to participate = 96,681  first survey page visitors = 159,301  Participation rate = minimum 60.7% (a student may have visited the page one or more times)  T2  visitors who agreed to participate = 30,311  first survey page visitors = 84,073  Participation rate = minimum 36.1% (a student may have visited the page one or more times) |
|  | Completion rate (Ratio of users who finished the survey/users who agreed to participate) | T1  users who finished = 68,891  users who agreed = 96,681  Completion rate = 71.3%  T2  users who finished = 22,540  users who agreed = 30,311  Completion rate = 74.4% |
| **Preventing multiple entries from the same individual** |  |  |
|  | Cookies used | No cookies were used. |
|  | IP check | No IP check was used. |
|  | Log file analysis | A unique identifier was defined for each participant based on 2 personal but non-identifying questions (name of preferred teacher, and last 5 digits of the phone number).  Duplicate database entries had the same user ID. In case of duplicate database entries, the full-filled questionnaire was preferred. If the same student had completed the entire questionnaire twice, the most recent version was kept for analysis. |
|  | Registration | No registration was used. |
| **Analysis** |  |  |
|  | Handling of incomplete questionnaires | Only students who completed all mental health outcomes were analyzed.  Note : At the end of the 3rd measurement time, the COSAMe database was frozen and the samples analyzed are those for which the data are complete. For the first 2 papers based on COSAMe, the sample analyzed corresponded to students for whom the data were complete only on the variables studied. The numbers of this article are therefore very slightly lower than those of the first 2 publications, without modifying the results. |
|  | Questionnaires submitted with an atypical timestamp | We did not define a minimal time to fill the questionnaire. |
|  | Statistical correction | Gender- and degree-standardized prevalence rates were calculated using the University Students population 2019-2020 published by the French Ministry of National Education (Ministère de l’Education Nationale de la Jeunesse et des Sports, 2020). |
